# Peptide-assembled nanoparticles targeting tumor cells and tumor microenvironment for cancer therapy

**DOI:** 10.3389/fchem.2023.1115495

**Published:** 2023-01-24

**Authors:** Meichen Zhang, Haiyan Xu

**Affiliations:** Institute of Basic Medical Sciences, Chinese Academy of Medical Sciences and Peking Union Medical College, Beijing, China

**Keywords:** peptide, nanoparticles, target, tumor microenvironment, cancer therapy

## Abstract

Tumor cells and corrupt stromal cells in the tumor microenvironment usually overexpress cancer-specific markers that are absent or barely detectable in normal cells, providing available targets for inhibiting the occurrence and development of cancers. It is noticeable that therapeutic peptides are emerging in cancer therapies and playing more and more important roles. Moreover, the peptides can be self-assembled and/or incorporated with polymeric molecules to form nanoparticles *via* non-covalent bond, which have presented appealing as well as enhanced capacities of recognizing targeted cells, responding to microenvironments, mediating internalization, and achieving therapeutic effects. In this review, we will introduce the peptide-based nanoparticles and their application advances in targeting tumor cells and stromal cells, including suppressive immune cells, fibrosis-related cells, and angiogenic vascular cells, for cancer therapy.

## Graphical Abstract

**Figure:**
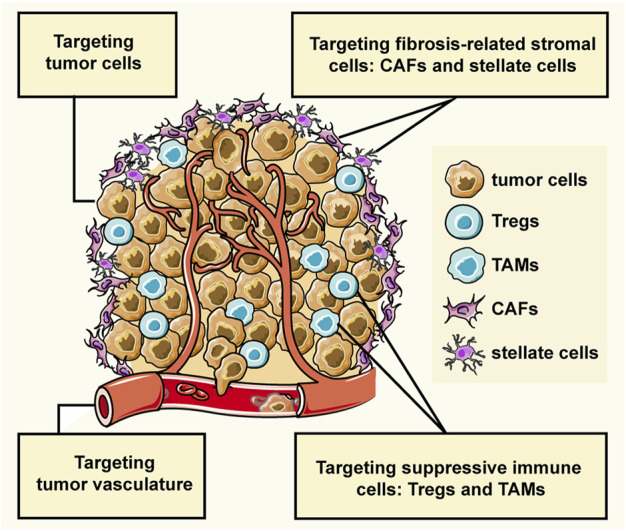
The interactions between tumor cells and stromal cells create the tumor microenvironment (TME) which largely affects the cancer progression. The stromal cells can be grouped into three general classes: angiogenic vascular cells, infiltrating immune cells, and tumor fibrosis-related cells. This review introduces the peptide-assembled nanoparticles targeting tumor cells and three types of stromal cells for cancer therapy.

## 1 Introduction

Tumor cells can recruit and corrupt normal cells, and the interactions between them create the tumor microenvironment (TME) that affects cancer progression (Balkwill et al., 2012; Arneth, 2019). Tumor cells can invade healthy tissues and spread to other parts of the body through the lymphatic or circulatory system. Non-malignant cells, namely stromal cells, usually promote the cancer progress by sustaining proliferative signaling, evading growth suppressors, resisting cell death, inducing angiogenesis, activating invasion and metastasis, evading immune destruction, and reprogramming energy metabolism (Hanahan and Coussens, 2012). In the hypothesis of “seed and soil”, tumor cells are considered as seed, and the microenvironment where the tumor cells live is soil that provides nutrition and survival guidance to the tumor cells, at the same time, the tumor cells educate the soil to become more friendly to the tumor cells (Paget, 1989). Stromal cells of the TME can be grouped into three general classes: angiogenic vascular cells, infiltrating immune cells, and tumor fibrosis-related cells (Hanahan and Coussens, 2012). Therefore, not only tumor cells but also the tumor microenvironment are available therapeutic targets in cancer treatment.

Up to date, some kinds of ligands have been developed for targeting tumor cells and stromal cells, such as antibody, peptide, and aptamer. In particular, peptides are emerging in cancer therapy in recent years and hold distinguished advantages compared with antibodies, such as low immunogenicity, structural variability, strong permeability, and more economic cost (Fosgerau and Hoffmann, 2015). Additionally, peptides can be carried by nanoparticles more easily than antibodies due to their low molecular weight and abundant amino residues, and nanoparticles-loaded peptide can be carried into cells as well as interact with the targeted proteins locating in the cell membranes.

Functional peptides can be classified into four categories, which are targeting peptides, microenvironment-responsive peptides, cell-penetrating peptides (CPP), as well as therapeutic peptides. However, due to its inherent shortcomings of easy aggregation and short half-life of circulating plasma, the application of peptides has been facing challenges of poor soluble stability *in vivo*, easy degradation by protease, and easy clearance by the kidney and liver (Zafar et al., 2021; Wang et al., 2022).

**FIGURE 1:**
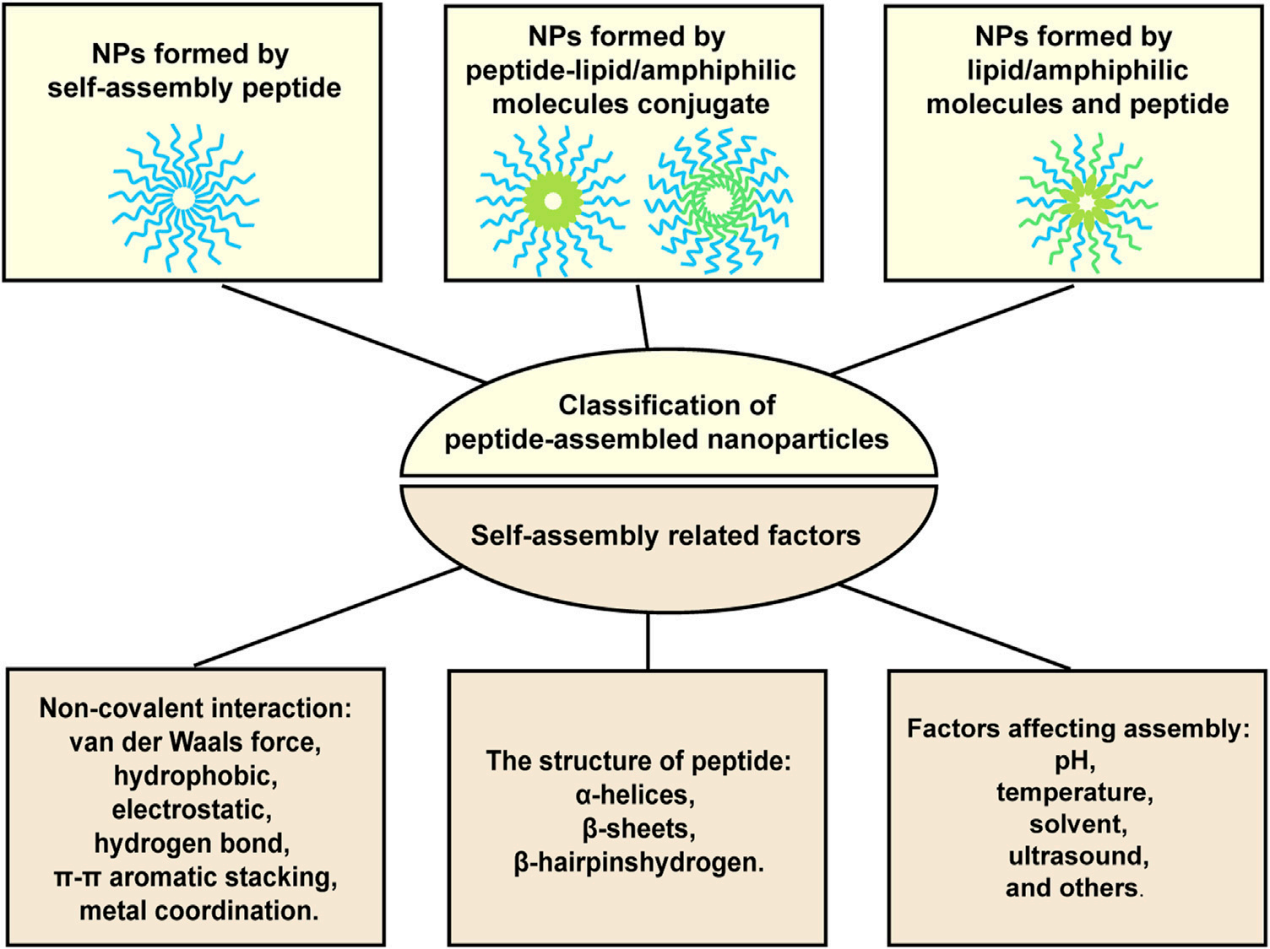
Illustration of the formation strategies for peptide-based nanoparticles.

It has been well documented that peptides themselves or with amphiphilic molecules, lipids, or hydrophobic drugs can be assembled into stable nanoparticles through intermolecular non-covalent interactions, including van der Waals force, hydrophobic, electrostatic, hydrogen bond, π-π aromatic stacking, and metal coordination. Factors affecting assembly include pH, temperature, solvent, ultrasound, and others. What’s more, secondary structure of peptide also determines the self-assembly occurs or not. The secondary structure preferring peptide self-assembly includes *a*-Helices, *ß*-Sheets, and *ß*-Hairpins (Yadav et al., 2020; Li et al., 2022). Figure 1 provided the illustration of the assembly strategies. By utilizing these assembly properties, peptide-based nanoparticles with designated functions can be fabricated. For examples, targeting peptides modified NPs can specifically bind to tumors and promote cellular uptake, thereby reducing off-target effects and improving anti-tumor efficacy. Microenvironment-responsive peptide-based NPs are pH-dependent release or only cleaved by enzymes in the TME, thereby reducing accumulation in normal tissues as well as causing toxic side effects. What’s more, the size of nanoparticles (NPs) is adjustable, and the appropriate particle size can not only avoid renal and hepatic clearance but also accumulate in the tumor site through the EPR effect (Chatterjee et al., 2014). More importantly, NPs can protect drugs from degradation and aggregation, and carry multiple therapeutic agents to achieve combined treatment and precisely resist tumor attacks in synchronize time and space manner (Yang et al., 2021).

Herein, we will describe the application of peptide-based nanoparticles that target tumor cells, suppressive immune cells, cancer-associated fibroblasts, stellate cells, and angiogenic cells in cancer therapies.

## 2 Therapeutic peptide-based nanoparticles targeting signaling proteins of the tumor cells

Tumor cells express tumor-associated protein that are low expressed in normal cells. These proteins can mediate tumor cells survival, proliferation, and migration through interacting with their ligands. Peptides can specifically bind to these protein targets, bring certain therapeutics to the tumor cells, or blocking the interactions between the proteins and their ligands (termed as to antagonist), which inhibit the activation of the down-stream signaling pathways. When antagonistic peptides are assembled with polymeric molecules and drugs, the peptide-based NPs combine the dual role of specifically targeting and antagonisms, which has showed promising potentials in the cancer treatment.

### 2.1 Targeting CXCR4

The C-X-C chemokine receptor 4 (CXCR4) is highly expressed in more than 23 kinds of human tumor cells and is a prognostic marker (Chatterjee et al., 2014). The interaction of CXCR4 and its ligand stromal-derived factor 1α (SDF-1α, also known as CXCL12) mainly secreted by stromal cells promotes several aspects of cancer progression including angiogenesis, metastasis, relapse, drug resistance, and survival (Teicher and Fricker, 2010; Mortezaee, 2020). Some peptide sequences targeting CXCR4 have been developed and investigated in various tumors, and some review articles have made a comprehensive summary. Here we will focus on those that are fabricated to nanoparticles by self-assembly and/or assembled with other molecules, as the nanoparticles can provide a powerful platform to load various drugs as well as the peptide, which extends the capacity of the peptides.

X4-2-6, a PEG-modified 24-amino acid peptide analog of the second transmembrane helix of CXCR4, could be self-assembled into homogeneous nanoparticles in which the PEG extensions at the C-terminus preventing superaggregation (Tarasov et al., 2011). The nanoparticles adopted predominantly a *ß*-Hairpin conformation in the aqueous solution, but folded into an *a*-Helix upon spontaneous fusion with the cell membrane. It was found that X4-2-6 peptide-based nanoparticles could inhibit CXCR4 function *in vitro* and hamper CXCR4-dependent bone metastasis of MDA-MB-231 cancer cells and prolong the survival in a mouse model of metastatic breast cancer. In addition, a hydrophobic drug HKH-40A was encapsulated by the nanoparticles with the *ß*-Hairpin conformation, and the drug was released when the nanoparticles encountered with the cells membrane, thus providing an interesting delivery system.

The conjugate of peptides and other fragments can be efficiently produced by genetic engineering, which can spontaneously self assemble into nanoparticles. A cationic peptide T22, a known antagonist of CXCR4, bound to and penetrated CXCR4^+^ cells efficiently *via* CXCR4-specific endocytosis (Nakashima et al., 1992). The researchers have constructed a CXCR4 targeted self-assembling nanoparticles by genetic engineering by combining peptide T22 with a polyhistidine H6 that has a strong dipolar charge distribution to support spontaneous self-organization (Céspedes et al., 2014). T22-GFP-H6 nanoparticles could effectively target and selectively internalize in several CXCR4^+^ tumor cells, such as colorectal cancer, diffuse large B cell lymphoma and head and neck squamous cell carcinoma tumor. Moreover, when administered in CXCR4^+^ tumor model, T22-GFP-H6 mostly accumulates in the tumor compared to other non-tumor bearing organs (Unzueta et al., 2012; Falgàs et al., 2020; Rioja-Blanco et al., 2022a). Furthermore, the active segments of the diphtheria toxin (DITOX) and the *Pseudomonas aeruginosa* exotoxin (PE24) were integrated with T22 and H6 to fabricate self-assembled toxin-based nanoparticles T22-DITOX-H6 and T22-PE24-H6, respectively, and they acted as toroid nanoparticles of 30–90 nm, penetrating the CXCR4^+^ tumor cells and promote tumor cell killing *in vitro*. *In vivo*, the nanocarriers mainly accumulated in tumor tissues (>75% of the administered dose) compared to non-tumor bearing organs, displaying a potent CXCR4-dependant antitumor effect in the absence of systemic toxicity in several CXCR4^+^ subcutaneous solid tumor mouse models (Sánchez-García et al., 2018; Serna et al., 2020; Voltà-Durán et al., 2021; Rioja-Blanco et al., 2022b; Falgàs et al., 2022). EPI-X4, a 16-residue fragment produced by proteolysis of human serum albumin, was the endogenous peptide inhibitor of CXCR4 (Zirafi et al., 2015). To form nanoparticles, the sequence was modified as EPIX4-(RK)-GFP-H6 that was efficiently produced in *Escherichia coli* by genetic engineering. The modified peptide was mixed with T22-GFP-H6 to form self-assembly biparatopic nanoparticles by divalent cation coordination through histidine-rich regions (Lee et al., 2011), which showed a dramatically improved biodistribution in the mouse models of CXCR4^+^ human cancer, faster cell internalization and enhanced cell killing effect when compared to the version based on a single ligand.

Our group reported a *de novo* CXCR4 antagonistic peptide (named as E5) (Li et al., 2014; Li et al., 2015; Guo et al., 2017) and prepared a micelle formulation by using amphipathic polymer DSPE-mPEG2000 through a one-step self-assembly method to improve the soluble stability of E5 in the physiological environment. M-E5 achieved effective therapeutic effects on a refractory AML mouse model (Meng et al., 2020). Moreover, a co-delivery system of doxorubicin (Dox) and E5 with DSPE-mPEG2000 was constructed (Zhang et al., 2022), which can effectively bind to the CXCR4-expressing AML cells, downregulate the signaling proteins mediated by CXCR4/CXCL12 axis and increase the cellular uptake of Dox. Importantly, M-E5-Dox can improve the blood circulation time and blood concentration of Dox, and enhance the delivery of Dox to AML cells infiltrating tissues and organs. M-E5-Dox remarkably decreases the proportion of leukemic cells in the peripheral blood, bone marrow, spleen, liver, and lung of the refractory AML mice, which in turn prolongs the survival significantly. In another study, M-E5 has a higher affinity for CXCR4 overexpressing human solid tumor cells and a stronger ability to inhibit CXCL12-induced tumor cell migration compared with E5 (Fang et al., 2017). Furthermore, it has been confirmed that lymph node metastasis can be a source of tumor cells for distant metastases in solid tumors which was related to CXCR4/CXCL12 axis (Pereira et al., 2018). E5 is too small to accumulate in the lymphatic system, while the PEG-micelles M-E5 with a diameter of 20 nm showed lymph node tropism and prevented metastatic tumor cells from colonizing the lungs (Fang et al., 2021).

It should be noted that peptides can also be combined with nanozymes to enable the nanozyme with targeting and antagonistic functions. For example, CXCR4 and CD44 dual-targeted Prussian blue nanosystem and Fe_3_O_4_@Pt nanozymes combined with CXCR4 antagonists E5 were developed (Bai et al., 2021; Kong et al., 2021). The combination of E5 and nanozymes displayed a superior synergistic therapeutic efficacy against AML *in vitro* and *in vivo*, preventing AML cells from homing to bone marrow and migrating to the spleen, lung and liver, which in turn prolonged the survival period of AML mice.

### 2.2 Targeting CD123

The interleukin-3 receptor alpha chain (IL-3Rα), named as CD123, is widely overexpressed in various hematological malignancies, especially in the leukemic stem cells and more differentiated leukemic blasts, which makes CD123 a promising therapeutic target. Several drugs have been developed to target CD123 for the treatment of leukemia, such as anti-CD123 antibodies (CSL-360, talacotuzumab and KHK2823), CD3 × CD123 bispecific antibodies (flotetuzumab, vibecotamab, JNJ-63709178, APVO436, and SAR440234), antibody-drug conjugates (SGN-CD123A), and anti-CD123 chimeric antigen receptor T cell immunotherapy, but none of them have crossed the clinical trial due to serious side effect, including severe myelosuppression, infusion-related adverse reactions, infection, hemocytopenia, cardiotoxicity, and gastrointestinal dysfunction (Slade and Uy, 2020; Espinoza-Gutarra et al., 2021).

Our group has chemically synthesized a novel antagonistic peptide PO-6 targeting CD123 by using a lab-developed cell-based selection process of a fragmental screening method, which is the first reported peptide targeting CD123 (Xu et al., 2021; Xu et al., 2022). To increase the antagonistic ability as well as to improve the soluble stability of the peptide in the physiological environment, PO-6 was mixed with an amphipathic polymer (Soluplus) through a one-step self-assembly method to form micelles (^m^PO-6) that were used as monotherapeutics in the animal model of refractory acute myeloid leukemia. Results showed that PO-6 could specifically bind to the CD123^+^ AML cells and the micellar formulation ^m^PO-6 increased the dissolution stability and the specific binding capacity. In the AE and C-KIT^D816V^ mouse model, ^m^PO-6 significantly reduced the percentage of AML cells infiltration and prolonged the median survival of AML mice by interfering CD123/IL-3 axis *via* regulating the activation of STAT5, PI3K/AKT, and NF-κB signaling pathways.

## 3 Targeting suppressive immune cells to enhance the anti-tumor effect

Immunosuppressive cells in TME are the main cause of tumor immune escape and are closely related to the poor prognosis of cancer patients. One of the current peptide-based immunotherapy methods is to synthesize peptide vaccines that are the same as tumor-associated antigens (TAAs) presented on the tumor cells to help cytotoxic T cells (CTL) produce a stronger immune response. However, this method is still limited by the downregulation of tumor cell TAAs and the instability of the peptide vaccine. It can be noticed there is another immunotherapy method that is to target immunosuppressive cells, such as tumor-associated macrophages (TAMs), regulatory T cells (Tregs) and myeloid-derived suppressor cells (MDSCs), to delivery therapeutic drugs. Among them, MDSCs is an immature cell group, and no specific biomarker of MDSCs for peptide binding has been found on its surface. In the following parts, we mainly introduced peptide-based self-assembled nanoparticles targeting Tregs and TAMs.

### 3.1 Targeting regulatory T cells

Regulatory T cells (Tregs) are a sub-type of CD4^+^ T cells which affect the activation of the immune system. Tregs inhibit the function of cytotoxic T cells by releasing cytokines, directly splitting and activating inhibitory receptors on the cell surface, thus promoting tumor immune escape in TME. Tregs are characterized by the expression of CD4, CD25, neuropilin-1 (NRP-1), and forkhead box P3 (FoxP3) (Bruder et al., 2004; Kelley and Parker, 2010).

Neuropilin-1 (NRP-1), a cell surface transmembrane glycoprotein, is regarded as the receptor for the class 3 semaphorins subfamily and vascular endothelial growth factor (VEGF) family members. NRP-1 is expressed on the majority of Tregs and promotes its activity, which was highly associated with Tregs-specific Foxp3^+^ expression (Bruder et al., 2004; Delgoffe et al., 2013; Glinka and Prud'homme, 2008). The tLyp-1 is a cell-penetrating peptide with a C-terminal R/KXXR/K consensus sequence, which has high affinity and specificity for NRP-1 receptor (Teesalu et al., 2009; Roth et al., 2012). The tLyp1 peptide-conjugated hybrid nanoparticles (tLyp-1-hNPs), incorporating tLyp-1-PEG-DSPE conjugate on the outer layer as the target ligand for the NRP-1 receptor and a PLGA core loading anti-CTLA-4 antibody and the tyrosine kinase inhibitor imatinib, was constructed by the oil-in-water (o/w) single-emulsion method (Ou et al., 2018). The tLyp-1-hNPs, which effectively targeted Tregs, presented good stability and enhanced suppressive effects against Tregs through inhibition of STAT3 and STAT5 phosphorylation and accumulation in Tregs. *In vivo*, tLyp1-hNPs mainly accumulated in the tumor areas *via* the enhanced permeability and retention (EPR) effect with decreased accumulation in the spleen and liver, owing to the suitable particle size, surface modification with DSPE-PEG and the NRP-1 targeted peptide, resulting in the significant reduction of the tumor volume and prolong of the survival of mice in the B16/BL6 tumor model.

### 3.2 Targeting tumor-associated macrophages

In different immune microenvironments, macrophages can display different phenotypes: M1 (anti-tumor) and M2 (tumor-promoting). Tumor-associated macrophages (TAMs) abundant in TME, which usually display as M2 type, are usually associated with poor prognosis in most cancers (Cassetta and Kitamura, 2018). TAMs play a tumor-promoting role by stimulating tumor inflammation, promoting angiogenesis, suppressing antitumor immunity, resulting in adaptive resistance, and accelerating tumor cell metastasis (Xiao and Yu, 2021). TAMs are becoming a key target of immunotherapy. Several TAMs-targeted peptides have been reported (Table 1), such as CD206-targeted peptides [RP-182 (Jaynes et al., 2020), melittin (Lee et al., 2017), UNO (Scodeller et al., 2017) and mUNO (Asciutto et al., 2019)], retinoid X receptor beta-targeted peptide [CRV (Tang et al., 2019)], vitamin D receptor-targeted peptide (CSSTRESAC), M2pep (Cieslewicz et al., 2013) and RVG (Zou et al., 2017).

**TABLE 1:** Peptides targeting TAMs.

Peptide	Sequence	Receptor	Identification	Advantage/Disadvantage	Ref
M2pep	YEQDPWGVKWWY	Unknown	Phage display *in vitro*	Targeted receptor was not identified	Li et al. (2020)
RP-182	KFRKAFKRFF	CD206	In silico biophysical homology screening	Also bound to RelB and CD47	Jaynes et al. (2020)
Melittin	GIGAVLKVLTTGLPALISWIKRKRQQ-NH2	CD206	In Lewis lung carcinoma mouse model	Had toxic to normal cells	Lee et al. (2017)
UNO	CSPGAKVRC	CD206	Phage display *in vitro*	Specifically targeted CD206 expressed on TAMs	Scodeller et al. (2017)
mUNO	(Linear version of UNO) CSPGAK	CD206	Phage display *in vitro*	Specifically targeted CD206 expressed on TAMs	Asciutto et al. (2019)
CRV	CRVLRSGSC, cyclic peptide	Retinoid X receptor beta (RXRB)	Phage display *in vitro*	RXRB may not be the surface marker of TAMs	Tang et al. (2019)
CSSTRESAC	CSSTRESAC	Vitamin D receptor	Phage display *in vitro*	Specifically bound to vitamin D receptor	Staquicini et al. (2021)
RVG	YTIWMPENPRPGTPCDIFTNSRGKRASNG	Nicotinic acetylcholine receptor (AchR)	Cell-based selection	AChR expressed on macrophage and microglia	Zou et al. (2017)

A peptide has been identified in 2013, termed M2pep (YEQDPWGVKWWY), which selectively bound to and internalized into M2-polarized TAMs as compared to M1 polarized mouse macrophages or other leukocytes (Cieslewicz et al., 2013). The CSF-1/CSF-1R pathway is crucial for the differentiation and survival of macrophages, providing a potential target of reprogramming TAMs from M2 to M1. PI3K-γ regulates a critical switch between immune stimulation and suppression and the blockade of PI3K-γ reprograms TAMs to stimulate CD8^+^ T cell-mediated tumor suppression and to inhibit tumor cell invasion and metastasis. Li et al. (2020) have constructed M2pep-modified self-assembly nanomicelles co-encapsulating CSF-1R-siRNA and small molecule PI3K-γ inhibitor NVP-BEZ 235 to target TAMs and modulate its polarization. The nanomicelles have enhanced TAMs targeting efficiency compared with non-targeting micelles both *in vitro* and *in vivo*. After intravenous injection, the dual drug-loaded nanomicelles suppressed pancreatic tumor growth by re-building tumor immune microenvironment, which transformed the M2-like TAMs into the M1-like phenotype and consequently enhanced CD8^+^ and CD4^+^ T cell infiltration. The dual pathway inhibition by the TAMs-targeting nanomicelles provided an alternative approach for cancer immunotherapy. Another TAMs targeting nanoparticles (M2NPs) were constructed with anti-CSF-1R siRNA, modified by *a*-Peptide (a scavenger receptor B type 1 targeting peptide) and M2pep (Qian et al., 2017). *In vitro*, it had a higher affinity to TAMs than to tissue-resident macrophages in the liver, spleen, and lung after being administered to B16 melanoma mice. Compared with the control treatment groups, M2NPs-based siRNA delivery resulted in the effective elimination of TAMs (52%), decreased tumor size (87%), and prolonged survival with superior biocompatibility. Additionally, this molecular-targeted strategy reprogramed the cytokine secretion and restored the function of infiltrating CD8^+^ T cells in the TME, providing a potential strategy of molecular-targeted cancer immunotherapy for clinical application.

RVG peptide, derived from a rabies virus glycoprotein, has been shown to bind specifically to macrophages *via* highly expressed AChR on the cell surface (Kim et al., 2010). In a study, self-assembly nanoparticles (RVG-PTX-NPs) were constructed by a modified nanoprecipitation method, with paclitaxel (PTX) encapsulated inside and RVG conjugated on the surface. RVG-PTX-NPs with desirable size (∼140 nm) showed poor uptake by neurons, while were able to cross BBB and selectively target and were preferentially internalized in brain TAMs with controlled release and tumor-specific toxicity, resulting in an effective therapeutic effect in the human glioma mice model (Zou et al., 2017).

## 4 Targeting fibrosis-related stromal cells to improve tumor microenvironment

Unlike tumor cells, stromal cell types within the TME are genetically stable and thus represent an attractive therapeutic target with reduced risk of resistance and tumor recurrence (Quail and Joyce, 2013). There are a lot of fibrosis-related stromal cells in the tumor microenvironment such as cancer-associated fibroblasts (CAFs), stellate cells, and adipocytes, which lead to special pathological TME with high fibrosis and excessive connective tissue hyperplasia. Adipose stromal cells, the precursors of adipocytes, induce epithelial-mesenchymal transformation (EMT), and adipocytes can transdifferentiate into CAFs (Bochet et al., 2013; Su et al., 2019). Stellate cells are precursors of CAFs in many solid tumors, which can also transform into CAFs through EMT (Yan et al., 2020a). CAFs can deteriorate the TME and densify the cancer matrix, and hinder drug penetration in tumors, playing a major role in promoting tumor progression and metastasis. The peptide-based NPs targeting adipocytes have rarely been reported, so we will focus on CAFs and stellate cells.

### 4.1 Targeting stellate cells

In solid tumors, pancreatic stellate cells (PSCs) and hepatic stellate cells (HSCs), the precursors of CAFs, are the major stromal cell type presenting in tumor tissues and the principal source of ECM production and fibrosis in the stroma which provides physical barriers to inhibit the distribution and penetration of various antitumor drugs (Apte et al., 2013). Peptide-based self-assembly nanoparticles targeted to stellate cells have been reported.

Cell-penetrating peptides (CPP, VSRRRRRRGGRRRR) with positive charge enables them to spontaneously form non-covalent nanocomplexes with negatively charged oligonucleotides *via* electrostatic interactions (Lee et al., 2013). CPP-based nanocomplexes were constructed to delivery anti-miRNA-199a oligonucleotides to primary human pancreatic stellate cells (hPSCs) (Schnittert et al., 2017). Results showed that the uptake of dimeric CPP-based nanocomplexes (NC-2) by hPSCs was 130 times higher than that of monomer-based nanocomplexes (NC-1). NC-2 was efficiently transfected into hPSCs by clathrin-mediated endocytosis compared with normal human fibroblasts and human pancreatic tumor cells. The delivery of anti-miR-199a inhibited hPSC differentiation into CAFs and inhibited the size of 3D heterospheroids composed of hPSCs and tumor cells.

PSCs and tumor cells overexpressed matrix metalloproteinases (MMPs), which in turn provided opportunities to design responsive materials for pancreatic tumor drug delivery (Yokota et al., 2002). A peptide with a sequence of Ac-CSSSGPLGIAGQSSS-COOH was synthesized, in which a MMP-2 specifically cleavable sequence “-GPLGIAGQ-” was incorporated. Based on it, a *ß*-Cyclodextrin (*β*-CD) modified matrix metalloproteinase-2 (MMP-2) responsive liposome (LRC-GEM-PFD) was constructed by a thin-film hydration method to regulate PSCs (Ji et al., 2016a), and RGD peptides were also modified onto the liposomes for targeting tumor cells. Pirfenidone (PFD), an antifibrotic and anti-inflammatory agent was inserted in the hydrophobic chamber of *ß*-CD, and the chemotherapeutic drug gemcitabine (GEM), was encapsulated in the liposomes. The liposomes were disassembled into two functional parts upon MMP-2 cleavage at the tumor site. The *ß*-CDs and PFD were kept in the stroma and inhibited the expression of collagen I and TGF-β in PSCs, down-regulating the fibrosis and decreasing the stromal barrier. The RGD peptide-modified-liposome loading GEM targeted and killed pancreatic tumor cells. Systemic administration of LRC-GEM-PFD into mice resulted in the alleviation of stromal fibrosis in pancreatic tumors and increased drug perfusion, ultimately showing an improved efficiency for pancreatic cancer therapy without overt side effects. MRP, another tailor-designed MMP-2 responsive amphiphilic peptide **(**sequence: SDK(C18)SGPLG-IAGQSK(C18)DS), tends to self-assemble to micrometer-long nanofibers. When it was mixed with the phospholipid, the co-assembly products transformed into stable hybrid liposomal nanospheres with uniform size distributions. Thus, a MMP-2 responsive peptide-hybrid liposome (MRPL) was constructed *via* co-assembly of phospholipids and MRP to deliver PFD to PSCs (Ji et al., 2017). The MRPL achieved tumor-specific delivery and release of PFD at the PSCs-enriched pancreatic tumors. The released PFD downregulated the multiple components of ECM secreted by the PSCs and increased the penetration of GEM into the tumor, thus enhancing the therapeutic efficacy of free GEM. Above all, the MMP-2 responsive nanomedicine may provide a potential strategy for improvement of the pancreatic cancer therapy.

### 4.2 Targeting cancer-associated fibroblasts

CAFs are one of the important types of stromal cells in solid tumor tissue, which highly express certain proteins, such as fibroblast activating proteins, αV-integrin family, and *a*-Smooth muscle actin (*α*-SMA). CAFs also secrete a series of factors and proteins, such as CXCL12 and Tenascin C, which promote tumor metastasis and densify the tumor matrix to build a barrier to prevent drug penetration. Peptide-based assembling NPs for targeting CAFs have been summarized in Table 2.

**TABLE 2:** Peptide-based assembling nanoparticles for CAFs.

Peptide	Sequence	Receptor	Nanoparticles	Function	Ref
DRGETGPAC	DRGETGPAC	FAP-α	F-SOS/DC/D NCs *via* the w/o/w double emulsion method	Depleted CAFs by the light assist; Remodeled TME; Improved the tumor penetration and anticancer efficacy *in vivo*	Yan et al. (2020b)
CAP	Ac-ATK(C18)DATGPAK(C18)TA-NH_2_	FAP‐*α*	Self-assembled micelles (CAP NPs) with hydrophobic drugs by hydrophobic interaction	Cleaved by FAP‐*α* and release Dox to CAFs and tumor cells; Disrupted the stromal barrier and enhanced local drug accumulation; Inhibted the growth of tumor	Ji et al. (2016b)
RGD	RGD	Integrin α_v_β_3_	RPM@NLQ	Inhibited matrix fibrosis; Enhanced the permeability of drugs	Duan et al. (2022)
TR	c (RGDfK) AGYLLGHINLHHLAHL (Aib)HHIL-NH_2_	Integrin α_v_β_3_	TR-PTX/HCQ-Lip by the thin film hydration method and the pH-gradient method	Recognized the integrin α_v_β_3_^+^ cells; Enhanced the cellular uptake; Inhibited stroma fibrosis; Killed tumor cells	Shi et al. (2015); Chen et al. (2019)
FH	FHKHKSPALSPVGGG	Tenascin-C	FH-SSLNav liposome by the thin film hydration method	Enhanced uptake and cytotoxicity; Targeted tumor stroma; CAFs depletion; Inhibited the growth of tumors	Kim et al. (2012); Chen et al. (2016)
			FH-NB-Dox nanobubble	Ultrasound contrast agent; Delivered more Dox into CAFs; Enhanced the Dox-induced eradication under ultrasound irradiation	Guo et al. (2019)
CFH	CFHKHKSPALSPVGGG	Tenascin-C	CFH/OM-L liposome by pH gradient method	Targeted and deactivated CAFs; Reversed the EMT process; Reduced collagen deposition	Guo et al. (2022)

#### 4.2.1 Targeting fibroblast activation protein-α

Fibroblast activation protein-α (FAP-α) is a transmembrane serine protein, which has been identified as a diagnostic marker to distinguish CAFs from normal fibroblasts.

Yan et al. (2020b) developed FAP-α-targeting peptide-modified, singlet oxygen (^1^O_2_)-sensitive, and Dox-loaded self-assembly nanoclusters (NCs) *via* a double emulsion method. After administration, the NCs specifically targeted CAFs, generated a large amount of ^1^O_2_ under the assistance of light irradiation which depleted CAFs resulting in stroma attenuation and degrading the shell of NCs to release small-sized, positively charged Dox-loaded NCs. The Dox-loaded NCs could efficiently penetrate deeper regions of tumors to release Dox which played a synergistic role with ^1^O_2_ in anti-tumor. This study provided a prospected way to promote the penetration of NCs in CAFs-rich tumors by simultaneously and spatiotemporally reconstructing the size and charge of NCs and remodeling TME. Other researchers constructed a FAP‐α responsive peptide-based nanoparticles (CAP NPs) to target CAFs and encapsulate Dox (Ji et al., 2016b). The cleavable amphiphilic peptide (CAP) specifically responsive to FAP‐α could self‐assemble into nanofibers, which would transform into drug-loaded spherical NPs when the hydrophobic drug Dox was added. *In vitro*, the Dox of CAP NPs could efficiently and specifically take up by CAFs, while normal cells did not. However, when CAFs were co-cultured with tumor cells, both cells have taken Dox, implying that the specific response of CAP NPs to FAP‐α could avoid off‐target effects and allow the released Dox to CAFs and the co‐existing tumor cells in TME. *In vivo*, the CAP NPs displayed outstanding tumor specificity and low non-specific organ accumulation. In the xenograft PC‐3 prostate tumor model, MCF‐7 breast tumor model, and Mia‐paca‐2 pancreatic tumor model, the CAP NPs were cleaved by FAP‐α and disassembled to release free Dox to disrupt the stromal barrier and enhance local drug accumulation resulting in the completed inhibition of cancers. This stimulus‐responsive nanocarrier was potentially applicable for the delivery of a broad spectrum of poorly soluble chemotherapeutic drugs, being able to greatly enhance tumor targeting and drug delivery efficacy. What’s more, some researchers have constructed self-assembled nanoparticles based on anti-FAP‐α antibodies and cell-penetrating peptides (CPP). Ji et al. (2015) designed and synthesized an amphiphilic peptide-cholesterol monomer (C2KG2R9) to deliver Dox to make CAFs depleted. The C2KG2R9 monomers, composed of CPP, oligo arginine and cholesterol, easily self-assembled to form positively charged core-shell NPs, in which hydrophobic Dox is encapsulated in the core, and anti-FAP‐α antibody is modified on the surface through electrostatic binding. After administration in nude mice co-implanted with CAFs and PC-3 cells subcutaneously, the NPs bound to CAFs, enhanced the uptake of Dox by CAFs and led to the depletion of CAFs and the destruction of matrix barrier, resulting in improving the penetration of Dox to tumor tissues.

However, the strategy of CAFs depletion is controversial, because rapid elimination of CAFs may break the homeostasis of the TME and increase the risk of metastasis (Özdemir et al., 2014). CAFs inactivation, rather than depletion, reduces the risk of tumor cell metastasis. CXCL12, mainly secreted by activated CAFs in desmoplastic tumors, maintained the tumor-promoting phenotype of CAFs, which accelerated tumor growth and metastasis. When CXCL12 expression is knocked down, CAFs are inactivated (Kojima et al., 2010). The researchers constructed cell-penetrating peptide-based nanoparticles to deliver CXCL12 silencing siRNA (siCXCL12) to CAFs by targeting FAP-α (Lang et al., 2019). In short, the conjugate of cholesterol and cell-penetrating peptide R9 (Chol-R9) self-assembled to form nanoparticles *via* hydrophobic interaction, then siCXCL12 and anti-FAP-α antibody were adsorbed to the positive-charged surface of NPs in turn *via* electrostatic interactions. The NPs specifically delivered siCXCL12 into CAFs by binding the FAP-α on the cell membrane of CAFs, knocked down the expression of CXCL12, resulting in the CAFs inactivation, the CAFs-related malignant TME remodeling and the inhibition of migration, invasion, metastasis, and tumor angiogenesis *in vitro*. In an orthotopic prostate tumor model, the NPs prolonged the blood circulation time and improved antimetastasis efficacy with no toxicity and acute immunological reaction. The TME reshaping strategy *via* targeting and inactivated CAFs instead of killing it provided an alternative approach for malignant prostate tumor metastasis inhibition.

#### 4.2.2 Targeting integrin

Integrin receptors are highly expressed in tumor cells and surrounding stromal cells, playing a role in promoting tumor progression.

Autophagy plays a significant role in pancreatic cancer, especially in activating CAFs. Chen et al. developed a multifunctional tandem peptide TH-RGD (TR) which consisted of integrin αvβ3-targeting cRGD peptide and the pH-sensitive TH peptide (Chen et al., 2019). Based on this, they constructed TR peptide-modified liposomes (TR-PTX/HCQ-Lip) loaded with hydroxychloroquine (HCQ), and paclitaxel (PTX) to inhibit the stroma fibrosis and tumor cell autophagy. The TR peptide-modified liposomes (TR Lip) had good targeting and permeability attributed to the electrostatic adsorption internalization caused by the positive charge under acidic conditions and the receptor-ligand interaction of integrin and peptide RGD *in vitro* and *in vivo*. TR-PTX/HCQ-Lip could effectively target and inhibit autophagy of pancreatic cells and CAFs, kill tumor cells and inhibit matrix fibrosis in allogeneic and *in situ* pancreatic cancer models.

Duan et al. (2022) developed an MMP-triggered dual-targeting co-assembly micelle-in-liposome NPs to sequentially deliver antifibrotic drugs quercetin (Que) and chemotherapeutic agents PTX to remodel fibrotic TME and enhance chemotherapy. The integrin αvβ3-targeting peptide RGD-modified and PTX-loading micelles and Que were co-encapsulated into MMP-sensitive liposomes, which were further modified with the NGR peptide binding to APN overexpressed on tumor vasculature. After administration in the 4T1/CAFs-bearing BALB/c mouse model, the NPs were aggregated at the tumor site through integrin αvβ3 and APN dual-targeting and disassembled into free Que and PTX-loaded micelles upon the cleavage of MMP in TME. Que entered CAFs and downregulated the expression of Wnt16 to inhibit fibrosis, which enhanced the permeability and killing ability of PTX-loaded micelles. The sequential delivery systems for fibrotic TME remodeling and chemotherapy potentiation may provide a promising therapeutic strategy for breast and other CAFs-rich tumors.

#### 4.2.3 Targeting tenascin C

Tenascin C is a tumor-specific extracellular matrix glycoprotein mainly secreted by CAFs, which is highly expressed in most solid tumors TME, but not in normal adult tissues, and promotes tumor progression (Orend and Chiquet-Ehrismann, 2006). Tenascin C may exist on the cell membrane of CAFs before secretion, which may provide the possibility of targeting CAFs.

A small peptide FH screened by phage display peptide library that specifically binds to Tenascin C and can be used to target CAFs in TME to deliver drug and enhance drug accumulation (Kim et al., 2012). In a study, the researchers used FH peptide as CAFs targeting ligand, conjugated with DSPE-PEG_2000_, and prepared tenascin C-targeting self-assembled liposomes (FH-SSLNav) loaded with Navitoclax, a Bcl-2 inhibitor, through the thin film hydration method (Chen et al., 2016). *In vitro*, FH-SSLNav showed more cellular uptake and toxicity. In Hep G2 tumor-bearing nude mice model, FH-SSLNav efficiently targeted tumor matrix and depleted CAFs to inhibit tumor growth. In another study, they constructed a CAFs-targeted and Dox-loaded ultrasonic nanobubble (FH-NB-Dox NB) consisting of the lipids as shell membrane, the FH peptide as CAFs targeting ligand and the perfluoropropan as core (Guo et al., 2019). FH-NB-Dox delivered more Dox into CAFs and enhanced the Dox-induced CAFs eradication under ultrasound irradiation and showed good contrast-enhanced ultrasound imaging *in vitro*. Another peptide targeting Tenascin C is the peptide CFH, which is obtained by connecting a cysteine at the end of the peptide FH (Guo et al., 2022). Based on it, a CAF targeting and oxymatrine-loaded liposome (CFH/OM-L) was developed by pH gradient method. CFH/OM-L could target and deactivate CAFs and reverse the EMT process accompanied by the upregulation of E-cadherin and downregulation of vimentin, N-cadherin, and snail protein *in vivo* and *in vitro*.

## 5 Targeting tumor vasculature

Angiogenesis plays a role in the growth, survival, metastasis and relapse of cancers. Angiogenic vascular cells, mainly endothelial cells, express specific molecular markers which are absent or barely detectable in the normal blood vessels, including integrins, aminopeptidase-N (APN or CD13), and VEGF receptors. Different from tumor cells, endothelial cells of tumor vasculature are genetically stable, and targeting it rather than tumor cells contributes to reducing drug resistance (Seyyednia et al., 2021). Therefore, tumor vasculature is a suitable site for cancer-targeted therapy. Most of the tumor vasculature-targeted peptides are also therapeutic peptides, which have the ability of anti-angiogenesis. The peptides targeting tumor vasculature are summarized in Supplementary Table S1.

### 5.1 Targeting integrin

Integrins are transmembrane-spanning receptors that play a critical role in tumor angiogenesis. RGD is the most classical peptide targeting integrin (Arap et al., 1998). Cyclized RGD is more stable and has a higher affinity (Kang et al., 2017). The tumor penetrating peptide iRGD synthesized by combining RGD with CendR motif not only binds to integrins αvβ3 and αvβ5, but also neuropilin-1 (NRP-1) (Kang et al., 2020; Li et al., 2021). Drug delivery systems based on RGD have been well summarized and will not be included in this review (Sadatmousavi et al., 2011; Danhier et al., 2012; Marelli et al., 2013; Sun et al., 2017; Sheikh et al., 2021a; Sheikh et al., 2021b; Cheng et al., 2021).

The C16Y peptide, which was derived from the laminin γ1 chain, bound to integrins αvβ3 and α5β1 strongly expressed on activated endothelial cells and tumor cells (Ponce et al., 2003). A study synthesized an amphiphilic chimeric peptide DEAP-C16Y that was able to self-assemble into spherical NPs at physiologic pH and dissociate to release individual peptides to target αvβ3 and α5β1 expressing cells in weakly acidic tumors (Ding et al., 2015). *In vitro*, DEAP-C16Y effectively inhibited endothelial cell migration, tubule formation and tumor cell invasion. In 4T1 breast cancer mouse models, the DEAP–C16Y NPs, with negligible cytotoxicity, were selectively located at the tumor site and suppressed tumor angiogenesis, growth, and metastasis. Through encapsulation of Dox, the DEAP-C16Y-Dox NPs have achieved more significant anti-tumor efficacy, due to the selective release and accumulation of Dox in tumor tissue. In another study, they prepared C16Y peptide-modified liposomes (C16Y-L) by a reverse-phase evaporation method, which could selectively target integrin αvβ3 and enhance intracellular uptake through energy-dependent endocytosis *in vitro* (Hamano et al., 2012).

AXT050, a collagen-IV derived peptide, bound to integrin αvβ3 with high affinity and had the ability to antitumor, anti-angiogenesis, and anti-lymphangiogenesis. An AXT050-modified PEGylated poly (lactic-co-glycolic acid) NPs was constructed (Bressler et al., 2018). The targeted NPs inhibited the adhesion and proliferation of tumor cells and endothelial cells through binding to integrin αvβ3 *in vitro* and showed two-fold greater accumulation in tumors and lower accumulation in the liver compared to non-targeted NPs in the triple-negative breast cancer mouse model.

Shroff et al. developed a fibronectin-mimetic peptide PR_b, which has been shown to bind specifically to integrin α5β1 (Shroff and Kokkoli, 2012). Based on this, they designed PR_b-functionalized PEGylated liposomes which could specifically bind to MDA-MB-231 cells through targeting integrin α5β1, and the binding could be controlled by varying the peptide concentration. The Dox-loaded liposomes were present in the early endosomes after 10 min incubation and in the late endosomes and lysosomes after long time incubation, which had equally cytotoxic as the free Dox, especially at higher doxorubicin concentrations. This study offered a promising strategy to deliver Dox to breast cancer cells.

### 5.2 Targeting aminopeptidase N

The enzyme aminopeptidase N (APN, also known as CD13) is related to angiogenesis, proliferation, invasion and metastasis, which is used as a diagnostic and prognostic factor for solid cancers (Wickström et al., 2011). NGR peptide was found to target APN by phage display in 1995 (Pasqualini et al., 1995). A tumor-penetrating peptide iNGR was screened out in 2013, composed of NGR motif and CendR motif (Alberici et al., 2013). iNGR initially bound to APN, with iNGR proteolytically cleaved to CRNGR, and then targeted NRP-1 to mediate deep penetration in the tumor parenchyma.

The major problem for glioma therapy is the poor extravasation and penetration of the nanodelivery system. The iNGR-modified nanodelivery system with the advantages of tumor blood vessel recognition and tumor penetration has been widely used in the treatment of glioblastoma, like iNGR-modified doxorubicin-loaded liposomes (Zhou et al., 2017), iNGR-modified PTX-loaded PEG-PLGA nanoparticles (Kang et al., 2014), and self-assembled RNAi nanoparticles functionalized with iNGR to siRNA delivery (An et al., 2015). All of them significantly facilitated the cellular uptake by HUVEC cells and glioma cells and enhanced the effect of drugs *in vitro*. In the glioblastoma-bearing mouse model, the iNGR-modified NPs displayed favorable pharmacokinetics, penetrated through tumor vasculature and into the deep tumor tissues and achieve the highest accumulation at the glioma sites.

iNGR-modified NPs also used in breast cancer for efficient drug delivery to tumor sites. In a study, the researchers constructed self-assembly ultrasmall micelles by the amphiphilic linear-dendritic PEG-PTX conjugate with a hydrated diameter of about 25 nm, which encapsulated PTX and linked with peptide iNGR (Zhang et al., 2017a). In triple-negative breast cancer mouse model, the micelles enhanced the accumulation in the tumor, displayed strong antitumor effect, and significantly extended the median survival time. In another study, the iNGR-modified Dox-loaded smart NPs with a PEGylated lipid monolayer shell and a pH-sensitive hydrophobic poly-l-histidine core was developed, which undergoed a two-step phase transition at two different pH values (Ye et al., 2018). In TME (pH_e_: 7.0–6.5), the surface potential of the smart NPs turned neutral or positive, facilitating the cellular uptake. After internalization, due to the acid endolysosome (pH_endo_: 6.5–4.5), the smart NPs dissociated and induced endolysosome escape to release Dox into the cytoplasm. The iNGR-modified smart NPs displayed higher cellular uptake *in vitro* and strongly inhibited tumor growth and prolonged the median survival time in late-stage aggressive breast carcinoma mouse model, providing a method for overcoming the problem of inefficient cellular uptake and intracellular drug release at the tumor site.

The angiogenesis-targeting iNGR peptide-modified TPGS-PLA nanoparticles co-encapsulating with chemotherapeutic agent docetaxel and photosensitizer verteporfin were constructed to treat drug-resistant human colorectal adenocarcinoma. The NPs promoted the cellular uptake and cytotoxicity of HUVEC cells and drug-resistant HCT-15 tumor cells and effectively inhibited tube formation under laser irradiation *in vitro*. In the drug-resistant HCT-15 mouse model, the NPs extravasated from the tumor vasculature and enhanced the inhibition of angiogenesis, selectively accumulated in the tumor site and penetrated deep site, and induced severe apoptosis and necrosis in tumor tissues under laser irradiation. It was evidenced that the NPs have great potential to treat drug-resistant tumors *via* efficient angiogenesis-targeted photo-chemotherapy.

### 5.3 Targeting VEGF receptor

Vascular endothelial growth factor (VEGF) and its receptors, such as VEGFR-1, VEGFR-2, and neuropilin-1 (NRP-1), have been implicated in pathological angiogenesis associated with tumors, intraocular neovascular disorders and other conditions, which were regarded as target molecules for the antiangiogenic approach (Ferrara et al., 2003).

Oku’s team has identified angiogenic vessel-homing peptide APRPG binding to VEGFR-2 and utilized it in liposomal drug delivery. They constructed VEGFR-2-targeted liposomes modified with PEG and DSPE-PEG-APRPG to deliver Dox, with the ability of long-circulating characteristics, enhanced accumulation in tumor and efficient tumor growth suppression *in vivo* (Maeda et al., 2004). Next, they developed dual-targeting liposomes modified with APRPG targeting VEGFR-2 and GNGRG targeting APN to deliver Dox. Compared to single-targeting liposomes, the dual-targeting liposomes were remarkably associated with human umbilical vein endothelial cells (HUVEC) and suppressed the growth of it *in vitro* and strongly suppressed tumor growth by the disruption of tumor vasculature in carcinoma-bearing mice (Murase et al., 2010). They also developed APRPG-modified liposomes encapsulating insoluble angiogenesis inhibitor SU5416, with the advantages of the improvement of solubility, the enhancement of antiangiogenic and the suppression of tumor growth with no remarkable side effects (Katanasaka et al., 2008). Guo et al. (2015) conjugated APRPG to the amphipathic copolymer PLGA-PEG to synthesized copolymer APRPG-PEG-PLGA which could spontaneously self-assemble to micelles and it was used to encapsulate PTX. The micelles promoted the uptake by endothelial cells and enhanced the cytotoxicity of cancer cells *in vitro*. After intravenous injection, the micelles accumulated in the tumor tissues due to the passive accumulation and active targeting effects, leading to reducing the tumor volume and prolonging the survival time of tumor-bearing mice.

K237, isolated from the phage display peptide library, was found to target VEGFR-2 predominantly expressed on the surface of tumor neovasculature endothelial cells with high affinity and specificity and induce the inhibition of angiogenesis and tumor growth (Hetian et al., 2002). Fang’s team developed K237-conjugated PTX-loaded nanoparticles (K237-PTX-NPs) for tumor neovasculature targeting drug delivery. *In vitro*, the K237-PTX-NPs could be significantly internalized by HUVEC by binding to VEGFR-2, thereby enhancing cytotoxicity and apoptosis. The K237-PTX-NPs with long-circulating property could accurately target tumor neovasculature, and induced the apoptosis of tumor neovasculature endothelial cells and necrosis of tumor tissues in MDA-MB-231 breast tumor-bearing mice (Yu et al., 2010). What’s more, the anti-tumor effect of K237-PTX-NPs is consistent with that of an 8-fold dose of free PTX plus P-gp inhibitor XR9576 in P-gp expressing human colorectal resistant tumor model (Bai et al., 2013). Yao et al. (2017) developed neovasculature and circulating tumor cells (CTCs) dual-targeting NPs encapsulating PTX, which is conjugated with K237 peptide and CTCs-targeted Ep23 aptamer. The dual-targeting NPs enhanced the cellular uptake, cell apoptosis, cytotoxicity and antiangiogenesis activity *in vitro* and actively captured and eradicated CTCs *in vivo*. The NPs damaged the primary tumor site and eradicated CTCs simultaneously and achieved a synergistic anti-tumor therapeutic effect.

A7R and proteolytically stable ^D^A7R, identified by phage display peptide library, showed high affinity for VEGFR-2 and NRP-1 and blocked VEGF-mediated angiogenesis and were commonly used as co-targeting ligands for anti-glioma targeted therapy (Binétruy-Tournaire et al., 2000). Several dual-targeted NPs have been reported, such as Dox and vincristine dual-loaded liposomes modified with ^D^A7R and glioma-targeted peptide T7 (Zhang et al., 2017b), Dox-loaded liposomes functionalized by ^D^A7R and nicotine acetylcholine receptor-targeting peptide ^D^CDX (Ying et al., 2016), and PTX-loaded nanoparticles modified with A7R and heparan sulfates-targeting peptide CGKRK (Hu et al., 2016), which could cross the blood-brain barrier (BBB) and blood-brain tumor barrier (BBTB) and displayed high glioma localization and strong antiangiogenesis and antiglioma effect.

### 5.4 Targeting nucleolin

Some studies show that nucleolin, known as a nuclear and cytoplasmic protein, can express on the cell membrane of activated tumor cells and tumor-specific vascular endothelial cells. A research group developed peptide F3 that specifically targeted to this new marker and mediated nucleolin-dependent internalization of drug loaded NPs. (Christian et al., 2003).

Treatment of glioma with NPs is limited by its low vascular permeability and tumor penetrability. tLyp-1, a tumor homing and penetrating peptide, was able to mediate tissue penetration through the NRP-1-dependent internalization pathway. Researchers constructed F3-modified PTX-loaded PEG-PLA NPs (F3-NPs) by an emulsion/evaporation method and co-administered with tLyP-1 to achieve tumor targeting along with high cellular internalization and extensive vascular extravasation (Hu et al., 2013a). Results showed that the F3-NPs could effectively penetrate 3D multicell tumor spheroids and increased the cytotoxicity of PTX *in vitro*. The F3-NPs co-administered with tLyp-1 showed the highest accumulation at the tumor site and deep penetration into the glioma parenchyma and achieved the longest survival in mice bearing intracranial C6 glioma.

The CCL5/CCR5 cytokine-related pathways in TME could regulate VEGF in a variety of ways, resulting in the ineffectiveness of tumor vascular normalization therapy dependent on anti-VEGF. In order to overcome this problem, the researchers combined two nanoparticles for administration focusing to achieve the bi-directional modulation between tumor vasculature and TME (Deng et al., 2021). One self-assembly nanoparticle (FLG NP) was constructed by an amphiphilic conjugate, composed of F3 peptide and two VEGFR-2 inhibitors connected with acid-sensitive hydrazone bonds. The FLG NP could specifically degrade and release VEGFR-2 inhibitors to repair abnormal tumor vessels when entering acidic conditions in VECs. Another nanoparticle loading with CCL5/CCR5 blocker maraviroc was designed to inhibit the CCL5 functions of angiogenesis and TME deterioration. Results showed that the combination of two NPs synergistically induced vascular normalization and remodeled TME in Panc-1 pancreatic cancer nude model and increased infiltration of CD4^+^ and CD8^+^ T cells in the B16-F10 melanoma model, providing a new direction for cancer therapy based on the tumor vasculature normalization and TME remodeling.

Wan et al. found that the F3 peptide-modified NPs enhanced accumulation in normal tissues resulting in serious side effects. Then they synthesized a pH-sensitive peptide CF, only displaying penetration ability under acidic conditions, by conjugating the tumor homing peptide CREKA with nucleolin-targeting peptide F3 through the acidic sensitive linker-hydrazone (Wan et al., 2019). Then the peptide CF was modified on the surface of the NPs co-entrapped with EGFR inhibitor EB and Notch inhibitor GSI-DAPT. Compared with other groups, the pH-sensitive NPs displayed the lowest distribution in normal tissues and the highest accumulation in the tumor site and strongly inhibited tumor growth in triple-negative breast cancer, due to the cell penetration ability of F3 was sealed under normal physical conditions and recovered at the acidic tumor environment in which pH-sensitive linkage can be broken down.

### 5.5 Other targeting peptides

In addition to the above mentioned, there are also other self-assembled nanoparticles based on tumor vasculature targeting-peptides for cancer treatment.

GX1, the cyclic 7-mer peptide motif CGNSNPKSC, was identified as a human gastric tumor vasculature homing peptide at first and bound to transglutaminase-2 (TMG2) to inhibit angiogenesis (Lei et al., 2018). The GX1-modified liposomes have been used to treat gastric cancer, such as GX1-modified liposomes encapsulating PTX (GX1-PTX-NLCs) and GX1-mediated anionic liposomes carrying adenoviral vectors integrating with the tumor suppressor gene of PTEN (GX1-Ad5-AL), which all promoted the cellular uptake by HUVEC cells, enhanced the inhibition of HUVEC cells and significantly suppressed the migration of gastric cancer cell *in vitro* (Xiong et al., 2015; Jian et al., 2020). *In vivo* studies demonstrated that GX1-PTX-NLCs significantly inhibited tumor growth with lower toxicity in MKN45 tumor-bearing nude mice model (Jian et al., 2020). GX1-conjugated poly (lactic acid) nanoparticles encapsulating Endostar, a novel recombinant human endostatin, and labeled with the near-infrared dye were developed for colorectal cancer targeting and therapy *in vivo*, which accumulated in tumor site and improved antitumor efficacy in the colorectal mouse model (Du et al., 2015).

E-selectin was exclusively expressed on the endothelial cells of tumor vasculature. The researchers designed a peptide (IELLQAR) to target E-selectin, and developed a PEGylated peptide-drug conjugate (PEGylated PDC) as a nano-prodrug, which was able to assemble into nanoparticles in aqueous conditions, by conjugating PEG and E-selectin targeting peptide to the antitumor molecule SN38 (Hao et al., 2021). The self-assembly NPs increased drug accumulation and retention at the tumor site by the EPR effect and improved tumor growth inhibition and prolonged the survival of mice bearing primary HCT116 tumors.

CGKRK peptide was specially bound to both neovascular endothelial cells and tumor cells and its receptor was reported to be heparan sulfate in 2007 (Järvinen and Ruoslahti, 2007) and mitochondrial protein p32 in 2013 (Agemy et al., 2013). CGKRK peptide functionalized and PTX loaded PEG-co-PCL NPs were developed by the emulsion/solvent evaporation technique, aiming to achieve tumor cells and tumor angiogenic blood vessels dual-targeting (Hu et al., 2013b). The dual-targeting NPs enhanced cytotoxicity and apoptosis on both HUVEC cells and U87MG cells and improved its inhibition effect on the growth of U87MG tumor spheroids *in vivo*. The dual-targeting NPs selectively accumulated at the tumor site and achieved the smallest tumor volume in mice bearing subcutaneous U87MG tumors. Above all, the tumor cells and tumor angiogenic blood vessels dual-targeting system might provide a great promising approach for reducing the disadvantages of antiangiogenic therapy alone.

IF7 peptide was designed to bind to the annexin 1 (Anxa 1), a novel specific biomarker of the tumor vasculature endothelial cells, with high affinity and specificity. IF7-PTX-NP nanoparticles modified with IF7 peptide and loading with PTX were developed by the emulsion/solvent evaporation method to treat resistant cancers (Yu et al., 2015). IF7-PTX-NP was significantly internalized by HUVEC through the IF7-Anxa 1 interaction and inhibited the tumor angiogenesis ability of HUVEC. IF7-PTX-NP prolonged circulation, accumulated in the tumor site, induced significant apoptosis of the tumor vascular endothelial cells and necrosis of the tumor tissues, and showed significant anticancer efficacy with a low dose (1 mg/kg) in the MCF-7/ADR xenografts in female nude mice. The same efficacy was only obtained with an 8-fold dose of PTX (8 mg/kg) or PTX plus P-gp inhibitor XR9576, which showed that targeting the tumor vasculature rather than the resistant tumor cells offered a promising strategy for the treatment of multidrug-resistant cancer.

The abundant presence of tumor vasculature and cancer stem cells (CSCs) promotes cancer metastasis. CD105 is a specific co-biomarker for tumor neovascular endothelial cells and CSCs, providing a potential target for inhibiting both cells simultaneously. An amphiphilic peptide based on the CD105 recognition motif was constructed, which could self-assemble into nanoparticles under physiological conditions (Wang et al., 2020). When nanoparticles bound to CD105, they transformed into nanofiber barriers on the cell membrane *in situ*, resulting in reducing endothelial permeability and angiogenesis and inhibiting the stemness and metastasis of CSCs in renal cancer. The NPs significantly inhibited the tumorigenesis and angiogenesis and reduced the metastatic nodules in lung in the patient-derived xenograft renal cancer mouse model, providing a promising method for inhibiting the metastasis of cancer.

## 6 Conclusion and prospect

Cancer is a multi-component entity, including tumor cells, stromal cells. This review described the advances of peptide-based nanoparticles in the cancer targeted therapies and summarized the currently existing peptides in tables. It should be noticeable that therapeutic peptides have evolved and played a notable role in the cancer treatment in recent years. Hence, it can be expected that peptide-assembled nanoparticles would provide alternative treatment strategies for cancer treatment in the future. Besides, peptide-drug conjugate*s* (PDCs) that combine therapeutic molecules with a peptide through a biodegradable linker are emerging and developed rapidly in recent years, which will become an appealing direction in the peptide field. It should be noted that most PDCs can self-assemble to different nanostructures, such as nanotubes, nanoparticles, and nanofibers, with the advantage of easy synthesis, alternative modes of administration, providing the means to tune the physicochemical properties, and a precise loading capacity (Wang et al., 2017; Fang and Wang, 2022).

Nevertheless, so far there have been no active-targeting carriers approved by FDA (Pérez-Herrero and Fernández-Medarde, 2015). Some concerns may be the potential blockades for successful translation of peptide-assembled nanoparticles into clinic. Firstly, tumor heterogeneity, multidrug resistance, inappropriate target and upregulation compensation in other ways may lead to the failure of targeted therapy. Secondly, the behavior of the peptide-based nanoparticles after entering the blood circulation cannot be completely tracked. Another issue is that all the transient metabolites and their potential toxicities should be identified and manifested, while there is no mature method to evaluate the biocompatibility and toxicity of peptide-assembled nanoparticles yet.

Above all, peptide-based assembling nanoparticles for cancer therapy has broad prospects and strong trends of sustainability.
