# Supplementary material for: Peptide-assembled nanoparticles targeting tumor cells and tumor microenvironment for cancer therapy
Source: Front Chem. 2023 Jan 24;11:1115495. doi: 10.3389/fchem.2023.1115495 (PMC9902599; doi:10.3389/fchem.2023.1115495)
Supplement: Supplementary file 1 [file Table1.docx]

Supplementary Material

**Table S1. Peptides targeting tumor vasculature**

| Peptide | Sequence | Identification | Receptor | Function | Ref |
| --- | --- | --- | --- | --- | --- |
| RGD | RGD | Phage display in vivo | Integrins αvβ3 and αvβ5 | Targeting tumor vasculature through binding to integrin. | (1) |
| iRGD | CRGDKGPDC | Phage display in vivo | Integrins αvβ3 and αvβ5; NRP-1 | The tumor targeting ability of iRGD is more intensified. | (2, 3) |
| cyclic RGD | c(RGDyC) | Phage display | Integrin αvβ3 | More stable and had higher affinity to integrin αvβ3. | (4) |
| TK | TWYKIAFQRNRK | Surface plasmon resonance analysis | Integrins α6β1 and αvβ3 | A targeted ligand for colon cancer. | (5, 6) |
| Oncothanin | CNYYSNSYSFWLASLNPER | A peptide from the alpha3 chain of type IV collagen | Integrin αvβ3 | Modified endothelial cell function and inhibited angiogenesis | (7) |
| AXT050 | LRRFSTAPFAFIDINDVINF | A systematic computational methodology based on bioinformatics | Integrin αvβ3 | Inhibited angiogenesis;  Anti-tumorigenic effects. | (8, 9) |
| PR_b | KSSPHSRN(SG)5RGDSP | Blocking experiments with peptides and antibodies | Integrin α_5_β_1_ | A targeted ligand for tumor vasculature. | (10) |
| Dabmaurin-1 | a Peptide Lebein-1-Like |  | A broad panel of integrins (αvβ6, αvβ3, αvβ5, α5β1, α6β4) | Exhibited anti-angiogenic effects at 30 nM in vitro. | (11) |
| C16Y | DFKLFAVYIKYR | Cell-based selection | Integrin αvβ3 and α5β1 | Inhibited angiogenesis. | (12, 13) |
| NGR | NGR | Phage display in vivo | APN | Inhibited angiogenesis. | (14) |
| iNGR | CRNGRGPDC | Phage display in vivo | APN and NRP-1 | Homed to tumor vessels and penetrated into tumor tissue more effectively than the NGR peptide. | (15) |
| CendR motif | (R/K)XX(R/K) X represents any amino acid | Phage display | NRP-1 | Mediated neuropilin-1-dependent cell, vascular, and tissue penetration. | (16) |
| tLyp-1 | CGNKRTRGC (containing CendR motif) | Phage display | NRP-1 and NPR-2 | Mediated tissue penetration through the neuropilin-1-dependent internalization pathway. | (17) |
| A7R | ATWLPPR | Phage display | VEGFR-2 and NRP-1 | Abolished VEGF binding to VEGFR-2; Inhibited the VEGF_165_ interactions with NRP-1; Decreased the tumor angiogenesis and growth. | (18, 19) |
| AS16 | ATWLPPRAANLLMAAS | Phage display in vivo | VEGFR-2 and Tie-2 receptors | Inhibited angiogenesis and tumor growth on sarcoma S180 and hepatoma H22 bearing BALB/c nude mice. | (20, 21) |
| APRPG | APRPG | Phage display in vivo | VEGFR-2 | A targeted ligand for tumor vasculature. | (22) |
| UPI | SGEEELQLQLALAMSKEEMGCIKSKRKCRGDKGPDC | Molecular modeling and binding studies | VEGFR-2 | Inhibited angiogenesis and tumor growth and increased survival rates in several tumor models. | (23, 24) |
| F56 | WHSDMEWWYLLG | Phage display | VEGFR-1 | Inhibited angiogenesis; Inhibited tumor growth and lung metastases in the breast cancer SCID mouse model. | (25) |
| K237 | HTMYYHHYQHHL | Phage display | VEGFR-2 | Disrupted the interaction between VEGF/VEGFR-2; Inhibited angiogenesis and tumor growth in breast cancer mouse model | (26, 27) |
| Esbp | DITWDQLWDLMK | Phage display | E-selectin | Delivered drug to the endothelial cells via binding to E-selectin. | (28) |
| IELLQAR | IELLQAR (mimic of E-selectin ligand) | Phage display |  | Inhibited sialyl Lewis X-dependent lung colonization of tumor cells. | (29, 30) |
| LRR5 | QMIVIELGTNPLKSSGIENGAFQGMK | Cell based-selection | Endothelial nitric oxide synthase (eNOS) | Inhibited angiogenesis; Inhibited the migration of endothelial cells. | (31) |
| LRR5C | SSGIENGAFQGMK | Cell based-selection |  |  |  |
| cavtratin | RQIKIWFQNRRMKWKKDGIWKASFTTFTVTKYWFYR | eNOS knockout mice model |  | Blocked microvascular permeability by inhibiting eNOS. | (32) |
| CGKRK | CGKRK | Phage display in vivo | p32 and heparan sulfates | A targeted ligand for tumor vasculature. | (33, 34) |
| AHKHVHVPVRL | AHKHVHVPVRL |  | CD105 | A self-assembly peptide targeting CD105. | (35) |
| NBD | Corresponding to MetAP2 (170–208) | Surface plasmon resonance analysis; In vitro binding assay | Prometastatic calcium-binding protein, S100A4 | Inhibition of angiogenesis in vitro and in vivo; Resulted in tumor regression in human prostate cancer xenografts. | (36) |
| SWL | SWLAYPGAVSYR | Phage display | EphA2 receptor | Targeted EphA2R and competed with ephrin ligands for binding. | (37) |
| YSA | YSAYPDSVPMMS |  |  |  |  |
| IF7 | IFLLWQR | Phage display | Annexin 1 (Anxa 1) | Suppressed tumor growth by conjugated to anticancer drug SN-38 in human colon HCT116 tumor nude mice model. | (38, 39) |
| RIF7 | RQWLLFI  (reverse sequence peptide of IF7) | Phage display |  | Increased stability and binding affinity compared to IF7. | (40) |
| TAASGVRSMH | TAASGVRSMH | phage display in vivo | NG2 | Specifically homed to tumor vasculature in wild-type tumor-bearing mice. | (41) |
| LTLRWVGLMS | LTLRWVGLMS |  |  |  |  |
| octreotide | FCFWKTCT (Disulfide bridge: Cys2-Cys7) |  | Somatostatin receptor subtype 2 (SST2) | Had angiostatic properties by itself. | (42) |
| F3 | KDEPQRRSARLSAKPAPPKPEPKPKKAPAKK | Phage display in vivo | Nucleolin | Accumulated in the nuclei of tumor endothelial cells and tumor cells. | (43, 44) |
| GX1 | a cyclic 7-mer peptide, CGNSNPKSC | Phage display in vivo | Transglutaminase-2 (TGM2) | Bound to the human gastric cancer vasculature; Inhibited angiogenesis. | (45, 46) |
| Gl-PP | Ganoderma lucidum Polysaccharide Peptide |  | Unkown | Inhibited angiogenesis. | (47) |
| rVBMDMP | rVBMDMP |  | Unkown | Inhibit angiogenesis. | (48) |
| FSEC | CELDENNTPMC |  | Unkown | Had anti-angiogenic and anti-tumorigenic effects in neuroblastoma. | (49, 50) |

**Reference**

1. Arap W, Pasqualini R, Ruoslahti E. Cancer treatment by targeted drug delivery to tumor vasculature in a mouse model. Science. 1998;279(5349):377-380. https://doi.rog/10.1126/science.279.5349.377

2. Li C, Chen Z, Zheng D, Zhao J, Lei J. Targeted Delivery of Dual Anticancer Drugs Based on Self-Assembled iRGD-Modified Soluble Drug-Polymer Pattern Conjugate Nanoparticles. ACS Appl Bio Mater. 2021;4(2):1499-1507. https://doi.rog/10.1021/acsabm.0c01388

3. Kang S, Lee S, Park S. iRGD Peptide as a Tumor-Penetrating Enhancer for Tumor-Targeted Drug Delivery. Polymers (Basel). 2020;12(9). https://doi.rog/10.3390/polym12091906

4. Kang W, Svirskis D, Sarojini V, McGregor AL, Bevitt J, Wu Z. Cyclic-RGDyC functionalized liposomes for dual-targeting of tumor vasculature and cancer cells in glioblastoma: An in vitro boron neutron capture therapy study. Oncotarget. 2017;8(22):36614-36627. https://doi.rog/10.18632/oncotarget.16625

5. Ren Y, Mu Y, Song Y, Xie J, Yu H, Gao S, Li S, Peng H, Zhou Y, Lu W. A new peptide ligand for colon cancer targeted delivery of micelles. Drug Deliv. 2016;23(5):1763-1772. https://doi.rog/10.3109/10717544.2015.1077293

6. Ren Y, Mu Y, Jiang L, Yu H, Yang S, Zhang Y, Wang J, Zhang H, Sun H, Xiao C, Peng H, Zhou Y, Lu W. Multifunctional TK-VLPs nanocarrier for tumor-targeted delivery. Int J Pharm. 2016;502(1-2):249-257. https://doi.rog/10.1016/j.ijpharm.2016.02.037

7. Shahan T, Grant D, Tootell M, Ziaie Z, Ohno N, Mousa S, Mohamad S, Delisser H, Kefalides N. Oncothanin, a peptide from the alpha3 chain of type IV collagen, modifies endothelial cell function and inhibits angiogenesis. Connect Tissue Res. 2004;45(3):151-163. https://doi.rog/10.1080/03008200490505923

8. Bressler EM, Kim J, Shmueli RB, Mirando AC, Bazzazi H, Lee E, Popel AS, Pandey NB, Green JJ. Biomimetic peptide display from a polymeric nanoparticle surface for targeting and antitumor activity to human triple-negative breast cancer cells. J Biomed Mater Res A. 2018;106(6):1753-1764. https://doi.rog/10.1002/jbm.a.36360

9. Lee E, Lee SJ, Koskimaki JE, Han Z, Pandey NB, Popel AS. Inhibition of breast cancer growth and metastasis by a biomimetic peptide. Sci Rep. 2014;4:7139. https://doi.rog/10.1038/srep07139

10. Shroff K, Kokkoli E. PEGylated liposomal doxorubicin targeted to α5β1-expressing MDA-MB-231 breast cancer cells. Langmuir. 2012;28(10):4729-4736. https://doi.rog/10.1021/la204466g

11. Chalier F, Mugnier L, Tarbe M, Aboudou S, Villard C, Kovacic H, Gigmes D, Mansuelle P, de Pomyers H, Luis J, Mabrouk K. Isolation of an Anti-Tumour Disintegrin: Dabmaurin-1, a Peptide Lebein-1-Like, from Daboia mauritanica Venom. Toxins (Basel). 2020;12(2). https://doi.rog/10.3390/toxins12020102

12. Kim H, Csaky KG. Nanoparticle-integrin antagonist C16Y peptide treatment of choroidal neovascularization in rats. J Control Release. 2010;142(2):286-293. https://doi.rog/10.1016/j.jconrel.2009.10.031

13. Ponce ML, Hibino S, Lebioda AM, Mochizuki M, Nomizu M, Kleinman HK. Identification of a potent peptide antagonist to an active laminin-1 sequence that blocks angiogenesis and tumor growth. Cancer Res. 2003;63(16):5060-5064.

14. Pasqualini R, Koivunen E, Kain R, Lahdenranta J, Sakamoto M, Stryhn A, Ashmun RA, Shapiro LH, Arap W, Ruoslahti E. Aminopeptidase N is a receptor for tumor-homing peptides and a target for inhibiting angiogenesis. Cancer Res. 2000;60(3):722-727.

15. Alberici L, Roth L, Sugahara KN, Agemy L, Kotamraju VR, Teesalu T, Bordignon C, Traversari C, Rizzardi GP, Ruoslahti E. De novo design of a tumor-penetrating peptide. Cancer Res. 2013;73(2):804-812. https://doi.rog/10.1158/0008-5472.Can-12-1668

16. Teesalu T, Sugahara KN, Kotamraju VR, Ruoslahti E. C-end rule peptides mediate neuropilin-1-dependent cell, vascular, and tissue penetration. Proc Natl Acad Sci U S A. 2009;106(38):16157-16162. https://doi.rog/10.1073/pnas.0908201106

17. Roth L, Agemy L, Kotamraju VR, Braun G, Teesalu T, Sugahara KN, Hamzah J, Ruoslahti E. Transtumoral targeting enabled by a novel neuropilin-binding peptide. Oncogene. 2012;31(33):3754-3763. https://doi.rog/10.1038/onc.2011.537

18. Starzec A, Vassy R, Martin A, Lecouvey M, Di Benedetto M, Crépin M, Perret GY. Antiangiogenic and antitumor activities of peptide inhibiting the vascular endothelial growth factor binding to neuropilin-1. Life Sci. 2006;79(25):2370-2381. https://doi.rog/10.1016/j.lfs.2006.08.005

19. Binétruy-Tournaire R, Demangel C, Malavaud B, Vassy R, Rouyre S, Kraemer M, Plouët J, Derbin C, Perret G, Mazié JC. Identification of a peptide blocking vascular endothelial growth factor (VEGF)-mediated angiogenesis. Embo j. 2000;19(7):1525-1533. https://doi.rog/10.1093/emboj/19.7.1525

20. Zhu X, Yang J, Gao Y, Wu C, Yi L, Li G, Qi Y. The dual effects of a novel peptibody on angiogenesis inhibition and M2 macrophage polarization on sarcoma. Cancer Lett. 2018;416:1-10. https://doi.rog/10.1016/j.canlet.2017.10.043

21. Wu D, Gao Y, Chen L, Qi Y, Kang Q, Wang H, Zhu L, Ye Y, Zhai M. Anti-tumor effects of a novel chimeric peptide on S180 and H22 xenografts bearing nude mice. Peptides. 2010;31(5):850-864. https://doi.rog/10.1016/j.peptides.2010.01.007

22. Katanasaka Y, Ida T, Asai T, Shimizu K, Koizumi F, Maeda N, Baba K, Oku N. Antiangiogenic cancer therapy using tumor vasculature-targeted liposomes encapsulating 3-(3,5-dimethyl-1H-pyrrol-2-ylmethylene)-1,3-dihydro-indol-2-one, SU5416. Cancer Lett. 2008;270(2):260-268. https://doi.rog/10.1016/j.canlet.2008.05.009

23. Dong Y, Wu H, Dong J, Song K, Rahman HA, Towner R, Chen H. Mimetic peptide of ubiquitin-interacting motif of epsin as a cancer therapeutic-perspective in brain tumor therapy through regulating VEGFR2 signaling. Vessel Plus. 2017;1:3-11. https://doi.rog/10.20517/2574-1209.2016.01

24. Dong Y, Wu H, Rahman HN, Liu Y, Pasula S, Tessneer KL, Cai X, Liu X, Chang B, McManus J, Hahn S, Dong J, Brophy ML, Yu L, Song K, Silasi-Mansat R, Saunders D, Njoku C, Song H, Mehta-D'Souza P, Towner R, Lupu F, McEver RP, Xia L, Boerboom D, Srinivasan RS, Chen H. Motif mimetic of epsin perturbs tumor growth and metastasis. J Clin Invest. 2015;125(12):4349-4364. https://doi.rog/10.1172/jci80349

25. An P, Lei H, Zhang J, Song S, He L, Jin G, Liu X, Wu J, Meng L, Liu M, Shou C. Suppression of tumor growth and metastasis by a VEGFR-1 antagonizing peptide identified from a phage display library. Int J Cancer. 2004;111(2):165-173. https://doi.rog/10.1002/ijc.20214

26. Bai F, Wang C, Lu Q, Zhao M, Ban FQ, Yu DH, Guan YY, Luan X, Liu YR, Chen HZ, Fang C. Nanoparticle-mediated drug delivery to tumor neovasculature to combat P-gp expressing multidrug resistant cancer. Biomaterials. 2013;34(26):6163-6174. https://doi.rog/10.1016/j.biomaterials.2013.04.062

27. Hetian L, Ping A, Shumei S, Xiaoying L, Luowen H, Jian W, Lin M, Meisheng L, Junshan Y, Chengchao S. A novel peptide isolated from a phage display library inhibits tumor growth and metastasis by blocking the binding of vascular endothelial growth factor to its kinase domain receptor. J Biol Chem. 2002;277(45):43137-43142. https://doi.rog/10.1074/jbc.M203103200

28. Shamay Y, Paulin D, Ashkenasy G, David A. E-selectin binding peptide-polymer-drug conjugates and their selective cytotoxicity against vascular endothelial cells. Biomaterials. 2009;30(32):6460-6468. https://doi.rog/10.1016/j.biomaterials.2009.08.013

29. Hao T, Fu Y, Yang Y, Yang S, Liu J, Tang J, Ridwan KA, Teng Y, Liu Z, Li J, Guo N, Yu P. Tumor vasculature-targeting PEGylated peptide-drug conjugate prodrug nanoparticles improve chemotherapy and prevent tumor metastasis. Eur J Med Chem. 2021;219:113430. https://doi.rog/10.1016/j.ejmech.2021.113430

30. Fukuda MN, Ohyama C, Lowitz K, Matsuo O, Pasqualini R, Ruoslahti E, Fukuda M. A peptide mimic of E-selectin ligand inhibits sialyl Lewis X-dependent lung colonization of tumor cells. Cancer Res. 2000;60(2):450-456.

31. Fan H, Sulochana KN, Chong YS, Ge R. Decorin derived antiangiogenic peptide LRR5 inhibits endothelial cell migration by interfering with VEGF-stimulated NO release. Int J Biochem Cell Biol. 2008;40(10):2120-2128. https://doi.rog/10.1016/j.biocel.2008.02.009

32. Gratton JP, Lin MI, Yu J, Weiss ED, Jiang ZL, Fairchild TA, Iwakiri Y, Groszmann R, Claffey KP, Cheng YC, Sessa WC. Selective inhibition of tumor microvascular permeability by cavtratin blocks tumor progression in mice. Cancer Cell. 2003;4(1):31-39. https://doi.rog/10.1016/s1535-6108(03)00168-5

33. Agemy L, Kotamraju VR, Friedmann-Morvinski D, Sharma S, Sugahara KN, Ruoslahti E. Proapoptotic peptide-mediated cancer therapy targeted to cell surface p32. Mol Ther. 2013;21(12):2195-2204. https://doi.rog/10.1038/mt.2013.191

34. Hoffman JA, Giraudo E, Singh M, Zhang L, Inoue M, Porkka K, Hanahan D, Ruoslahti E. Progressive vascular changes in a transgenic mouse model of squamous cell carcinoma. Cancer Cell. 2003;4(5):383-391. https://doi.rog/10.1016/s1535-6108(03)00273-3

35. Wang L, Lv Y, Li C, Yang G, Fu B, Peng Q, Jian L, Hou D, Wang J, Zhao C, Yang P, Zhang K, Wang L, Wang Z, Wang H, Xu W. Transformable Dual-Inhibition System Effectively Suppresses Renal Cancer Metastasis through Blocking Endothelial Cells and Cancer Stem Cells. Small. 2020;16(40):e2004548. https://doi.rog/10.1002/smll.202004548

36. Ochiya T, Takenaga K, Asagiri M, Nakano K, Satoh H, Watanabe T, Imajoh-Ohmi S, Endo H. Efficient inhibition of tumor angiogenesis and growth by a synthetic peptide blocking S100A4-methionine aminopeptidase 2 interaction. Mol Ther Methods Clin Dev. 2015;2:15008. https://doi.rog/10.1038/mtm.2015.8

37. Koolpe M, Dail M, Pasquale EB. An ephrin mimetic peptide that selectively targets the EphA2 receptor. J Biol Chem. 2002;277(49):46974-46979. https://doi.rog/10.1074/jbc.M208495200

38. Hatakeyama S, Sugihara K, Shibata TK, Nakayama J, Akama TO, Tamura N, Wong SM, Bobkov AA, Takano Y, Ohyama C, Fukuda M, Fukuda MN. Targeted drug delivery to tumor vasculature by a carbohydrate mimetic peptide. Proc Natl Acad Sci U S A. 2011;108(49):19587-19592. https://doi.rog/10.1073/pnas.1105057108

39. Hatakeyama S, Shibata TK, Tobisawa Y, Ohyama C, Sugihara K, Fukuda MN. Tumor targeting by a carbohydrate ligand-mimicking peptide. Methods Mol Biol. 2013;1022:369-386. https://doi.rog/10.1007/978-1-62703-465-4_28

40. Chen X, Fan Z, Chen Y, Fang X, Sha X. Retro-inverso carbohydrate mimetic peptides with annexin1-binding selectivity, are stable in vivo, and target tumor vasculature. PLoS One. 2013;8(12):e80390. https://doi.rog/10.1371/journal.pone.0080390

41. Burg MA, Pasqualini R, Arap W, Ruoslahti E, Stallcup WB. NG2 proteoglycan-binding peptides target tumor neovasculature. Cancer Res. 1999;59(12):2869-2874.

42. Starkey JR, Pascucci EM, Drobizhev MA, Elliott A, Rebane AK. Vascular targeting to the SST2 receptor improves the therapeutic response to near-IR two-photon activated PDT for deep-tissue cancer treatment. Biochim Biophys Acta. 2013;1830(10):4594-4603. https://doi.rog/10.1016/j.bbagen.2013.05.043

43. Christian S, Pilch J, Akerman ME, Porkka K, Laakkonen P, Ruoslahti E. Nucleolin expressed at the cell surface is a marker of endothelial cells in angiogenic blood vessels. J Cell Biol. 2003;163(4):871-878. https://doi.rog/10.1083/jcb.200304132

44. Porkka K, Laakkonen P, Hoffman JA, Bernasconi M, Ruoslahti E. A fragment of the HMGN2 protein homes to the nuclei of tumor cells and tumor endothelial cells in vivo. Proc Natl Acad Sci U S A. 2002;99(11):7444-7449. https://doi.rog/10.1073/pnas.062189599

45. Chen B, Cao S, Zhang Y, Wang X, Liu J, Hui X, Wan Y, Du W, Wang L, Wu K, Fan D. A novel peptide (GX1) homing to gastric cancer vasculature inhibits angiogenesis and cooperates with TNF alpha in anti-tumor therapy. BMC Cell Biol. 2009;10:63. https://doi.rog/10.1186/1471-2121-10-63

46. Lei Z, Chai N, Tian M, Zhang Y, Wang G, Liu J, Tian Z, Yi X, Chen D, Li X, Yu P, Hu H, Xu B, Jian C, Bian Z, Guo H, Wang J, Peng S, Nie Y, Huang N, Hu S, Wu K. Novel peptide GX1 inhibits angiogenesis by specifically binding to transglutaminase-2 in the tumorous endothelial cells of gastric cancer. Cell Death Dis. 2018;9(6):579. https://doi.rog/10.1038/s41419-018-0594-x

47. Cao QZ, Lin ZB. Ganoderma lucidum polysaccharides peptide inhibits the growth of vascular endothelial cell and the induction of VEGF in human lung cancer cell. Life Sci. 2006;78(13):1457-1463. https://doi.rog/10.1016/j.lfs.2005.07.017

48. Wang C, Cao J, Qu J, Li Y, Peng B, Gu Y, He Z. Recombinant vascular basement membrane derived multifunctional peptide blocks endothelial cell angiogenesis and neovascularization. J Cell Biochem. 2010;111(2):453-460. https://doi.rog/10.1002/jcb.22735

49. Taleb M, Atabakhshi-Kashi M, Wang Y, Rezvani Alanagh H, Farhadi Sabet Z, Li F, Cheng K, Li C, Qi Y, Nie G, Ying Z. Bifunctional Therapeutic Peptide Assembled Nanoparticles Exerting Improved Activities of Tumor Vessel Normalization and Immune Checkpoint Inhibition. Adv Healthc Mater. 2021;10(12):e2100051. https://doi.rog/10.1002/adhm.202100051

50. Chlenski A, Guerrero LJ, Peddinti R, Spitz JA, Leonhardt PT, Yang Q, Tian Y, Salwen HR, Cohn SL. Anti-angiogenic SPARC peptides inhibit progression of neuroblastoma tumors. Mol Cancer. 2010;9:138. https://doi.rog/10.1186/1476-4598-9-138
